# Ten simple rules for navigating the reference letter seeking process

**DOI:** 10.1371/journal.pcbi.1010102

**Published:** 2022-05-26

**Authors:** Courtney Peña, Latishya J. Steele, Debra S. Karhson, Judith T. Ned, Crystal M. Botham, Miranda B. Stratton

**Affiliations:** 1 Stanford Biosciences Grant Writing Academy, Stanford University, Stanford, California, United States of America; 2 Office of Graduate Education, Stanford University, Stanford, California, United States of America; 3 Psychology Department, University of Texas Permian Basin, Odessa, Texas, United States of America; 4 School of Medicine Human Resource Group, Stanford University, Stanford, California, United States of America

## Introduction

Letters of recommendation, also called reference letters, are a qualitative metric used to evaluate a candidate during an application process. The letters are written on behalf of a candidate by professionals such as an advisor, mentor, or supervisor. Letters of recommendation are required for additional training experiences like graduate/medical school, grants, fellowships, and other academic awards. Reviewers use the letters to vet a candidate and assess their potential for success. Reference letters are required at nearly every stage of the academic life span and have a significant impact on one’s ability to advance professionally [1,2]. For early-stage letter seekers such as undergraduate/graduate students and postdoctoral fellows, the norms and standards of the letter seeking process are rarely clear or defined. Fortunately, as a letter seeker, you have some agency in ensuring the quality and timeliness of your letters of recommendation [3–5]. This paper addresses common challenges that arise in the letter seeking process and offers 10 simple rules to guide letter seekers as they navigate the process of securing a strong letter of recommendation [3]. The 10 simple rules outlined below will be most useful for those who are seeking reference letters that are at least 2 months from the submission deadline. These rules can be modified depending on the letter seeker’s timeline and the nature of your application.

### Rule 1: Thoroughly read the application website and establish a timeline

Familiarize yourself with the application website so you understand what the organization is looking for in a successful applicant. Look closely for any requirements or eligibility criteria about letter writers. Application websites will often provide specific information about letter writers such as how many letters are required, the submission format, relationship to letter writer (i.e., supervisor, principal investigator, advisor, mentor, and faculty member), and how long they have known you. Carefully review application websites also to establish a timeline for when the letters of recommendation are due. Once you have an idea of the requirements and timing, start mapping out when you will contact your letter writers to ensure that they will have enough time (1 to 3 months depending on the nature of the application [3,5]) to write and submit the letter.

### Rule 2: Identify multiple potential letter writers

Using the criteria from the application materials that you gathered in Rule 1, brainstorm potential letter writers. One strategy for letter seekers—particularly helpful for students—is to create a Who do I know [6] table to think through your options. An example table is modeled below (Table 1). You can find a downloadable version of this resource here [6].

**Table 1:** Use the Who do I know table to identify potential letter writers.

Potential letter writer’s name	Nature of relationship	Depth and length of relationship	Why this person?	Asking? Yes/No/Alternate
Dr. X	Dissertation advisor	Close mentor for 5+ years: We meet regularly	Published together, has written supportive letters on my behalf	Yes
Dr. Y	Chair of department	Met at a conference, have not talked to since	Influential in their field, has mentored former awardees	No
Dr. Z	Former supervisor	Worked in their lab for 3 months during undergrad	Accessible and supportive; less familiar with current work	Alternate

A Who do I know table should include important information about what the application calls for, specific qualities about each potential letter writer, the nature of your relationship, how long you have known them, whether they are familiar with your work, and what materials could help them to write a strong letter. This will guide you in identifying which of the potential letter writers are the best fit for your application. Think strategically about the number of letter writers as well. For example, if an application calls for 3 to 5 letters of recommendation, try to secure at least 4 letter writers. That way, if one of your letter writers drops out, then you still have the minimum required letters, and your application isn’t impacted.

### Rule 3: Select letter writers that fit the criteria

Once you have finished your Who do I know table (Table 1), it should be clearer as to which individuals are most capable of writing a strong letter of recommendation on your behalf. If you are unsure about who to ask, consider talking through your table with a trusted peer or mentor to get their feedback. Select the potential letter writers that align the closest with the requirements and make sure these are people you know and that you are in good standing with them. It is not recommended to ask somebody who does not know you or your work. Strong letter writers will be able to speak to your potential for success because they are familiar with you and your work [5,7].

### Rule 4: Prepare to ask

Depending on your application, you may need multiple letters of recommendation. If that is the case, consider preparing an asking plan for each individual letter writer. As you prepare to ask for a strong letter of recommendation, position yourself in a way that reflects your preparedness and consideration for the letter writers’ time. It is important to think strategically about how you will contact your selected letter writers [5,7]. For example, will you contact them by email? Phone? During a scheduled meeting? Whatever it is, make sure you are contacting them at an appropriate time that works for them (i.e., not on major holidays, after work hours, weekends, or other times where they are not likely to be available). Have a proposed timeline ready to share with your letter writers with letter due dates, website links, and other logistics clearly indicated. Craft a compelling request that includes your shared work and why it makes sense for them to write the letter on your behalf.

Consider sharing materials, including drafts, which will help your letter writer compose a strong letter of recommendation that is consistent with the rest of your application [5]. For example, you can provide your CV or resume and an abstract or statement about your research or candidacy for the opportunity at hand. Share a list of bullet points that summarizes: your qualifications, relevant accomplishments, goals for the application, and how you have worked with the recommender in the past [5,7]. This list, sometimes known as a “brag sheet” [7], is a helpful resource for letter writers as it summarizes important points they can include in their recommendation and allows you some agency in how you will be represented in the letter. Make sure to also think about how you wish to be written about including your pronouns (see [8,9]) and preferred name. Clarify what your letter writers do and do not have permission to say about you in the letter both professionally and personally. Along with the materials above, include important links such as the website of the organization you are applying to, the application portal where they will submit the letter, as well as the submission deadline.

### Rule 5: Ask for a strong letter

Be specific about the kind of letter you want. Asking specifically for a strong letter invites the letter writer to reflect on their knowledge of you and the goal of the letter. When you ask for a strong letter, it opens up an opportunity for a conversation and what they can say about you with confidence. In this way, it is less likely that a letter writer who has reservations about writing a strong letter will agree to do so.

At this point, you have put a lot of thought into your letter seeking process. Being well prepared and organized during the asking stage will speak to your professionalism. Demonstrate your preparedness while you ask for the letter by providing your letter writers with the materials you prepared in Rule 4 (i.e., your summarized bullet points, application materials, links to the application’s website, etc.). Showing up prepared and demonstrating your thoughtfulness during this stage of the process could impact the letter writer’s decision to work with you on this and future applications.

Some letter writers may request you write a joint letter [4]. If this opportunity is presented to you, take it. Depending on the nature of your relationship with the letter writer, consider offering this as an option during the ask. Cowriting a letter provides you the opportunity to reflect on and assess your skills and abilities, agency to highlight specific examples that address the reviewed criteria, and gain practice in writing compelling and professional letters of recommendation [4] (see [3,10–12] for tips on writing effective reference letters that you can share with your letter writer). Additionally, given the ample research documenting racial/ethnic and gender bias in letters of recommendation [1,13–19], which contribute to the many disparities present in academia [20–23], cowriting your letter is one way to reduce the potential for gendered and/or biased language (see Avoiding Bias for Letters of Recommendation [12] and Ten simple rules for writing compelling recommendation letters [3] for tips on avoiding racial/ethnic and gender bias) or other errors in your letter which could negatively influence the reviewers [3].

If the person declines to write a letter, use your Who do I know table from Rule 2 to identify alternative letter writer(s). Once you have secured your letter writers, discuss how and when you will communicate with them during the writing process (see Rule 6).

### Rule 6: Establish a communication plan

As with any interpersonal relationship, strong communication is key. Having clear norms and expectations about how and when you will communicate on the progress of the letter will make the letter seeking process easier for everyone involved.

Take some time to get to know what works best for your letter writers as you want to demonstrate you value their time. During your conversation, check in with your letter writers about their preferred contact methods and frequency. Ask your letter writers: How can I keep us both accountable for meeting our shared goal of submitting this application on time? It is likely your letter writer will know what works for them and once you learn their preferences, you can establish a communication plan that you both agree on. This plan can serve as your accountability road map for you and your letter writers.

### Rule 7: Manage your letters

Follow through on your communication plan which you established in Rule 6. This will ensure that the letter writer is keeping this task on their radar, and you are following through on your end. Generally, you can track letter submissions through an application portal. The most important point here is that you are following your timeline and communicating as needed. At this stage in the process, consider the practice of mentoring up, also known as managing up, [24,25] where the mentee takes an active lead in the mentorship process. Mentoring up equips you with the tools and strategies to navigate this stage of the letter seeking process as you grow professionally.

### Rule 8: Have a backup plan

Sometimes, things don’t go as planned. It’s best to be prepared for any scenario. If a letter writer is suddenly unable to write a letter, return to your Who do I know table (Rule 2) to identify an additional letter writer. If a letter writer is not following up with the communication plan you set in place, you may need to contact an alternate in case they do not submit their letter by the deadline. For this reason, it is wise to have more than the minimum letter writers, if allowable, as you never know if someone will drop out. Sudden changes can best be mitigated by early and strategic planning (Rule 6). If you are getting stuck, connect with a trusted mentor on how to best move forward with submitting your application.

### Rule 9: Ensure that letters are received on time

Track the letters through the application portal and flag any that are missing. A good strategy is to ask your letter writer what they want you to do in case they have not submitted 48 hours from the deadline. Make sure you have discussed this ahead of time so that there are no surprises. Build this into your communication plan and timeline. It may be the case that you have to make a phone call or follow up with a firm reminder. If you are not able to secure the letters by the deadline, contact the organization and ask them if they can give an extension for a late letter.

### Rule 10: Maintain rapport after the application process

After your application is submitted, thank your letter writers for their contributions. An appropriate thank you would be a handwritten note or a follow-up email, but do not send gifts. Update your letter writers once you hear back about the status of your application. Your letter writers have contributed to your application and will likely be invested in the outcome [5]—even if it’s not the news you had hoped for. In any case, use the letter seeking process as a professional development experience in building relationships with mentors and potential colleagues. Maintaining strong rapport with your letter writers will make future collaborations easier and more pleasant.
